# Alternating Attention and Physical Fitness in Relation to the Level of Combat Training

**DOI:** 10.3390/healthcare10020241

**Published:** 2022-01-27

**Authors:** Dariusz Jamro, Grzegorz Zurek, Maciej Lachowicz, Dariusz Lenart, Malgorzata Dulnik

**Affiliations:** 1 Department of Physical Education and Sport, General Tadeusz Kosciuszko Military University of Land Forces, 51-147 Wroclaw, Poland; dariusz.jamro@awl.edu.pl (D.J.); dariusz.lenart@awl.edu.pl (D.L.); 2 Department of Biostructure, Wroclaw University of Health and Sport Sciences, 51-612 Wroclaw, Poland; maciej.lachowicz@ideis.pl (M.L.); mal.gosia009@gmail.com (M.D.)

**Keywords:** physical fitness, alternating attention, combat training, cadets

## Abstract

The level of combat training (CT) of the future commander-leader is of critical importance to the armed forces in national defense. This study aimed to search for the relationship between the level of alternating attention, physical fitness and shooting accuracy (SA), and academic achievements in practical military subjects (PMS). The study group consisted of 137 cadets of the Military University of Land Forces. The measure of alternating attention in the study was the Color Trails Test results. Motor components were assessed by measuring hand static strength, endurance run, and time of a speed and agility run. SA and PMS were taken as measures of cadets’ CT. Significantly higher PMS were associated with higher levels of strength and better endurance in cadets. The physical fitness of the cadets did not significantly affect the cadets’ SA. The main result of the study is the revelation of the level of alternating attention as a strong determinant of cadets’ SA. The authors suggest that the main emphasis should be put on the physical preparation of a modern soldier, focused on the development of strength and endurance skills. It is also reasonable to introduce cognitive stimulation exercises to shooting training.

## 1. Introduction

Armed forces are the basic element of the state defense system, designed for effective implementation of defense and security policies. They take part in the process of stabilizing the international situation, in crisis response and humanitarian operations, supporting internal security and helping society. One of the main elements of evaluating the efficiency and effectiveness of the army is its combat training (CT), which is treated as the ability to perform tasks in the armed forces under conditions of war and peace. The elements of CT consist primarily of the areas of tactical training, shooting, physical fitness (PF), medical training, commanding, topography, battlefield medicine, and mobilization capabilities. CT in peacetime demonstrates the readiness of an army to conduct direct combat in the event of an armed conflict or other crisis, where it will be necessary to use troops. Criteria for the evaluation of CT vary from one army to another and are determined depending on the organizational concept of the armed forces, the level of general and technical culture of the population and the state of health [1]. Despite the current technological progress, modern methods of warfare invariably require a great psychophysical effort from the soldier, so one of the main elements in the evaluation of CT is mental preparation and good PF [2,3,4].

Nevertheless, important determinants of CT and soldier’s readiness to conduct military operations are psychosocial factors, the key to which is a high level of motivation of soldiers and high morale. Most tasks and orders are externally motivated because military service is closely associated with subordination, hierarchy, control, devotion to regulations and rules. This indicates the important role of external motivation for soldiers. However, internal motivation plays an important role as it drives action even when external motivation ceases. Therefore, it is important for soldiers to feel satisfaction from the action itself, to see the importance of being given orders, to be able to make sacrifices, and even those connected with risking their own health and life. A study by Ryan and Deci (2000) identified three major psychological needs—competence, autonomy, and relationships—which, when satisfied, provide increased self-motivation and mental health. A high level of motivation is extremely important for both rank and file soldiers and command staff to mobilize not only themselves, but more importantly others for commitment, effort, and quality performance [5].

This corresponds with the contemporary concept of health-related fitness, where PF is considered a measure of the body’s ability to function efficiently and effectively, to stay healthy, and as a measure of the ability to maintain resilience and cope with crisis situations [6]. In contrast, as Howley and Franks (1977) write, “The goal of PF is positive physical health, which determines low risk of health problems. Performance, on the other hand, aims at the ability to engage in daily tasks with adequate energy and to participate satisfactorily in selected sports” [7].

Combat readiness has always been associated with PF testing in the army. Today, each unit in the armed forces creates its own standard based on different tasks and responsibilities [2]. To perform combat tasks effectively, military personnel need adequate levels of muscular strength, power, agility, coordination, and endurance [8,9,10].

In addition to physical and mental fitness, shooting training of each soldier is an extremely important determinant of CT. It boils down primarily to effective and accurate shooting at targets with individual weapons (pistols, carbines, selective fire rifles, etc.). Shooting performance may be affected by both physiological and psychological factors [11,12]. Shooting tasks are classified as a complex combat ability that requires the individual to perform multiple motor and cognitive tasks simultaneously (multitasking) or alternate (task switching) between tasks to achieve a given goal [13,14]. Additionally, shooting requires focused attention on the target as well as full control of coordination between postural activity and arm elevation (in the standing shooting stance) [15]. The results of Tremayne and Barry (2001) confirm that both attentional processing and motor preparation are involved in skillful pistol shooting [16]. At the same time, it should be noted that shooting accuracy (SA) largely depends on shooting experience and training [17].

Alternating attention (AA) is one aspect of attention. It is the ability to quickly change attentional focus and shift concentration deliberately from one direction (task, object, activity, etc.) to another without processing all the information coming to us [18]. It is manifested in practice by the ability to possibly shift one’s focus from performing one task to another [19], as well as the ability to focus attention and re-engage it in response to external stimuli [18]. AA is examined, among other things, by tests involving tracking and object finding such as the Color Trails Test (CTT) [20], which is a cross-cultural version of the neuropsychological Trail Making Test (TMT).

Analyzing the subject literature, according to our current knowledge, there is a lack of scientific papers in which direct relationships between AA and PF and CT of soldiers (cadets) have been investigated. Therefore, the purpose of this research is to try to find the relationship between the level of AA and PF and CT (SA and academic achievements in practical military subjects (PMS)) in a group of the Military University of Land Forces (MULF) cadets. Thus, the present study appears to be original and novel and its results should be of interest to those interested in military science, national security, shooting, cognitive and PF.

## 2. Materials and Methods

The study group consisted of cadets—first-year students of MULF in Wroclaw. Cadets are high school graduates who, after successful recruitment to MULF, were called to active military service as candidates for professional officers. While on active military duty, cadets study and receive their education at MULF as a Command Officer Candidate. As a result of 5-year studies, graduates are prepared to perform tasks in military units, organize and conduct training and educational activities at the platoon level. Cadets at the end of training are promoted to the rank of second lieutenant—the first officer rank of the Polish Army.

**Figure 1:**
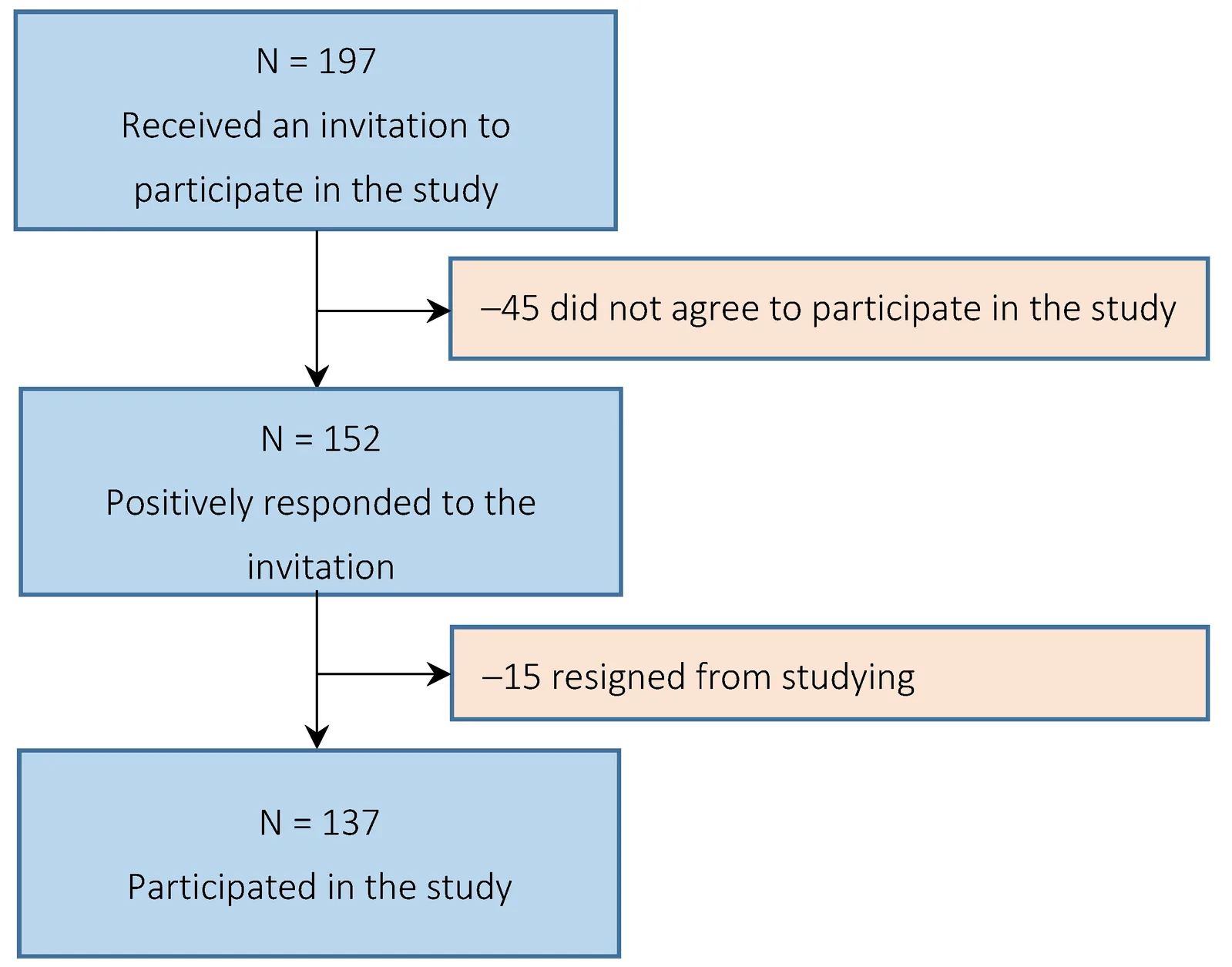
Flowchart of participant enrolment.

The criterion for inclusion in the study was obtaining promotion for the third semester of study and agreeing to informed participation in the study. Initially, 152 men meeting the inclusion criteria qualified for the study. During the course of the study, 15 cadets dropped out of the study by opting out of further military service, wanting to work in another field in the future. Finally, the complete results of 137 cadets were analyzed (Figure 1). The average age of the subjects was 21.03 years. The study was conducted in July 2021 at MULF.

The study was designed based on the health-related fitness (H-RF) concept in which health promotion and concern for functional performance and well-being play a key role. Attention was paid to those components of PF that primarily inform health i.e., cardiorespiratory and neuromuscular fitness and muscular strength [6].

### 2.1. Items by Which Motor Components Were Assessed

- Dominant hand static strength—hand muscle strength was measured using a Stanley hydraulic hand dynamometer. This is a precise instrument for measuring hand grip strength. The adjustable handle has five settings, allowing you to adjust the device to any hand, regardless of size, measuring up to 90 kg. The measurement was taken with an accuracy of 1 kg. The subject had to clench his hand as hard as he could. The result was read from the hand of the dynamometer, which stopped at the highest reading until zero. The test was performed twice and better result was taken for analysis.
- The time of the speed and agility run (10 × 5 m shuttle run)—the test was performed in a sports hall. After assuming the starting position in front of the designated line, after the command “START” the examined person ran as fast as they could to the second line, 5 m away, crossing it with both feet and returning. During the run, and especially during the turns, the starters could not support themselves with their hands on the floor. The test was performed once. The time was measured with an accuracy of 0.01 s. Time was measured with a professional hand-held Casio digital stopwatch with an accuracy of 0.01 s from the “START” command to crossing the final line after completing 10 repetitions of the designated 5-m sections.
- Endurance—the endurance run took place in an athletics stadium. At the signal “READY” the tested person stood behind the starting line in the high starting position. At the signal “START” the participants ran the fastest possible pace to the finish line for 2.5 laps (1000 m). The test was performed in 10-person groups. Time was measured with an accuracy of 1 s.

The study was always conducted by the same team consisting of experienced specialists in human motor skills. The examination was preceded by a warm-up, demonstration, and verbal commentary. PF was assessed based on a test developed by the International Committee for Standardization of Physical Fitness Test (ICSPFT) [21]. PF tests were always performed at the same time of day and in the same place. One day was allocated separately for each PF test, the tests were conducted in the following order: static strength of the dominant hand, 10 × 5 m shuttle run, 1000 m endurance run. Every effort was made to ensure that each test subject had similar testing conditions.

### 2.2. Alternating Attention (AA)

The measure of AA in the study was the results of the CTT, which is a paper-and-pencil neuropsychological tool designed to test adults aged 18 years and older.

The high validity and reliability of the CTT, as well as the simplicity of its tasks and procedure, combine to make it a particularly valuable tool for diagnosis in clinical psychology, particularly neuropsychology [20,22,23,24].

The CTT consists of two inseparable parts: CTT-1 and CTT-2, administered to the subject immediately one after the other. Each part contains colored (pink and yellow) circles in which numbers from 1 to 8 (trial) and 1 to 25 (test proper) are placed. In CTT-1, all odd numbers are printed on pink circles and all even numbers are printed on yellow circles. In CTT-2, each number is printed twice, once on a yellow background and once on a pink background. The test task is arranged on a sheet of white paper measuring 21.59 × 27.94 cm, with the trial task on the first side of the sheet and the main task on the reverse side [25].

The subject’s task was to connect the numbers in straight lines, without taking the pencil off the paper, in ascending order, with the numbers to be connected in such a way as to maintain alternating colors. The beginning and end of the task were marked with graphic symbols. The accuracy of the task and its duration were recorded [22].

Taking into account the differences in the CTT-1 and CTT-2 constructs, the CTT-2 results were used for this study because, unlike the CTT-1, where performance time is a measure of visual search, sustained attention, and graphomotor ability, the CTT-2 results additionally provide information about the divisibility of attention and AA and the ability to process information sequentially. In the context of the CT components examined, the CTT-2 results provide more valuable data.

According to D’Elia and colleagues, performance time indices of the CTT are used to measure functions related to the work of the frontal lobes of the brain. The test is used to examine a variety of processes related to attention and executive functions, specifically assessing intentional material search, sustained and AA, sequential information processing, and monitoring of one’s own behavior [20].

The CTT test was administered by a psychologist and took place individually in a lecture room, always under the same conditions and at the same time of day. The time of task completion was measured to the nearest 1 second. The correctness and evaluation of CTT-2 performance was checked twice.

### 2.3. The Measure of Cadets’ Combat Training (CT)

- Shooting accuracy (SA)

This included the results of two shots at a fixed target; the first consisted in firing from a carbine at a target 100 m away in a lying down shooting stance. The second event involved firing a military pistol at a target 15 m away in a standing stance. The shooter had five rounds and was required to shoot single fire. The maximum number of points it was possible to obtain was 50. The marksmanship score was the average of the points obtained from the two shooting attempts.

Shooting under the above conditions is primarily used to learn to shoot accurately with the least possible projectile scatter on the target. These tests are usually performed as first tests. They are the initial training for the performance at a later stage of advanced dynamic shooting, with changes of stances, in motion, and under a regime of time and other restrictions. All subjects had the same shooting experience resulting from the same MULF training program. The shootings always took place in similar weather conditions and at the same time of day.

b. Academic achievements in PMS, determined by the average grade at the end of the second semester of training in subjects:
  - Tactics,
  - military topography,
  - physical education.

The grades obtained by students ranged from 2–5, in half grade increments. The mean PMS score was the average of the final semester grades in the three subjects mentioned above

The explanatory variables were SA (mean score) and PMS (mean score in the subjects). PF components (handgrip strength, speed-agility running, endurance) and attention-related variable (CTT-2) were treated as explanatory variables in this study.

The study was based on the approval of the Rector—Commandant of the MULF (no. 271 dated 18 January 2021) and the consent of the Research Ethics Committee of the Wroclaw University of Health and Sport Sciences (no. 2/2021 dated 12 February 2021). All procedures performed in this study involving human participants were in accordance with the 1964 Helsinki declaration and its later amendments or comparable ethical standards. Written informed consent was obtained from all participants included.

## 3. Statistical Analysis

The collected results were statistically analysed by calculating for all variables: normality of distribution of individual variables was assessed by the Kolmogorov–Smirnov test, relationships between explanatory and explained variables were assessed by Pearson correlation, and relationships between AA and motor components of PF and cadets’ SA were analysed by progressive stepwise regression [26,27]. The explanatory variable in the regression analysis was SA (shooting scores), while the explanatory variables were: time achieved in the CTT-2 Color Trails Test, dominant hand grip strength, 10 × 5 m shuttle run time, and 1000 m run time.

Relationships between AA and motor components of PF and PMS were also calculated using progressive stepwise regression. The explanatory variable was PMS, while the explanatory variables were time achieved in the CTT-2 Color Trails Test, dominant hand grip strength, 10 × 5 m shuttle run time, and 1000 m run time. However, none of the explanatory variables entered the PMS prediction model, so the description of the results of this analysis is omitted in the following sections of the paper. The use of the regression method allowed us to determine the optimal set of explanatory variables (including interdependencies between them) that would best predict the performance of the explanatory variable—cadet SA. We chose to include in the model all four explanatory variables analyzed in the study because of the skills desired in military service (strength, endurance, running speed, and agility) and high levels of attention and concentration. Statistical significance in the study, for all tests used, was taken at *p* < 0.05.

All calculations were performed using Statistica v. 13 software from StatSoft in the Laboratory of Biostructure Research of the Wroclaw University of Health and Sport Sciences, certified according to ISO 9001.

## 4. Results

The basic descriptive statistics of the study group are shown in Table 1. It is noticeable that the results of the dominant hand grip strength test, endurance run, and CTT have the largest standard deviation.

An analysis of the simple relationships between levels of AA and PF with SA and PMS are presented in Table 2. Higher SA was significantly favored by higher AA of cadets. Of note is the relatively high Pearson correlation coefficient (−0.57) indicating a strong relationship.

Among the PF tests, a significantly positive correlation occurred between the dominant hand grip and PMS and a significantly statistically negative correlation between the 1000 m run and cadets’ PMS. Significantly higher PMS was associated with higher levels of strength and better endurance in cadets. However, the strength of these correlations was weak.

The results of the regression analysis (using the progressive stepwise method) are presented in Table 3. Finally, only one variable was significantly statistically included in the predictive model of SA—the results of the CTT-2 Color Trails Test (β = −0.566). The level of AA, examined by the above test, was revealed as a strong determinant of SA, which significantly explains 31.5% of the variability in cadets’ SA (adjusted R^2^ = 0.315).

## 5. Discussion

There is growing evidence in the literature of the positive relationship between PF, physical activity and aerobic training and academic achievement and more efficient brain functioning. It has been confirmed in human and animal studies that physical activity, particularly aerobic activity, can have a positive impact on many aspects of brain function and cognition. It can therefore be inferred that higher endurance capacity may contribute to higher academic performance. However, these studies, unlike our own, were conducted on older adults and on animals [28].

In other studies, PF as a construct including attributes related to health and ability has been linked to academic performance in children and adolescents. Of a total of 45 studies included, 25 reported a positive association between PF components and academic performance [29]. Physical activity has been shown to have a positive effect on concentration, memory, intellectual performance, and behavior in primary grades [30], although other studies among secondary school children and adolescents have not found significant correlations. However, a significant limitation of this study was the questionnaire-based assessment of physical activity. Academic performance was assessed by exam scores in English, mathematics, and science, among others [31]. Although the results of the above studies correspond with the results of our own study, it is difficult to make direct comparisons due to the different study groups and the different nature of academic achievement. To the best of our current knowledge, there is a lack of research in the literature on the determinants of academic achievement of student-cadets, where the measure of such achievement is in practical subjects.

The cadets’ level of PF expressed in static strength and endurance had a significant relationship with academic achievement in PMS which, along with SA, speak to their level of CT. In contrast, AA was not significant for academic achievement in PMS. Perhaps this is related to the practical nature of the academic achievements included in the study. Indeed, different conclusions spring from the work of Alavi et al. (2019), who found that academic performance in theoretical subjects increased as attention levels increased. However, it should be noted that this study was conducted in a different age group [32].

The results of this study confirm that PF is highly important and desirable among future commanders. Similarly, as indicated by previous studies of Norwegian cadets, the level of endurance and strength of soldiers is of particular importance, ensuring safe and effective performance of physical military work [33]. In this study, a relationship was sought between the results of selected PF tests (push-ups, squats, pull-ups, standing medicine ball throw, and Sargent’s jump) and skeletal muscle mass, as a physiological factor responsible for generating maximum muscle power (strength). This factor, in turn, directly affects the effective performance of combat tasks indicative of CT such as evacuation of the wounded, crawling, marches with loads, rapid changes of direction.

Despite the development of modern technologies in military operations, the duties of soldiers constantly consist of tasks requiring high physical effort, such as carrying or lifting heavy loads and materials, prolonged physical effort with additional load and combat equipment, dynamic tactical training or combat shooting. In a study by Pihlainen et al. (2014), the average work intensity in the measured military tasks (e.g., weighted marches, lifting and carrying weights ranging from 10 to 43 kg, digging out a shooting position while lying down), was close to 50% of the soldiers’ maximal aerobic capacity, which suggests, similar to the results of our study, that the level of fitness is one of the key components of PF affecting the level of CT. However, it is difficult to directly compare the results of the cited studies with our own results due to the use of different research tools. Additionally, the study was conducted in a group of conscript soldiers and not cadets [8].

In order to effectively perform combat tasks, military personnel need adequate levels of muscular strength and endurance and additionally power, agility and coordination. A study by Kraemer and Szivak (2012) highlights the key role of anaerobic strength training for the demands of the modern battlefield [9]. Strength and endurance exercises are ideally suited for the use of functional actions and movements that reflect combat tasks. Military commanders recognize the need for combat-focused physical training, i.e., programs that target the tasks that can be expected during combat [10,34]. Based on the results of our research, the authors suggest directing the physical preparation of the modern soldier towards the development of strength and endurance capabilities. It seems that less attention can be paid to the running speed and agility of soldiers.

Researchers from the U.S. military community correctly note that standard military PF tests do not actually test what is needed on the battlefield. In these tests, the main emphasis is on testing endurance levels, while battlefield tasks also require strength [35]. These observations coincide with the results of our work, where, in addition to endurance, strength also significantly correlated with elements of CT of soldiers.

In our study, cadets’ SA was not related to their motor fitness. In other authors’ studies the effectiveness of shooting was observed after anaerobic exercise [35], overcoming obstacle courses [36] as well as after marching with loads [37]. Upper body movements occurring during breathing significantly impaired the subjects’ ability to keep the weapon on target. Moreover, the fatigue of the upper body made it much more difficult to shoot effectively in a standing position. The authors of this study rightly observed that strenuous physical exertion clearly decreases shooting efficiency. Therefore, it should be assumed that the initial higher PF may contribute to the maintenance of the SA level after the physical effort, through a quick recovery after the effort and a smaller decrease of the fitness. Thus, the lack of relationship between SA and motor fitness of cadets from our study may be due to the fact that the shooting was static and not preceded by physical exercise.

Making an attempt to interpret the results, according to our current knowledge, it is necessary to mention the lack of similar studies of the relationship between cognitive processes (including AA) and the level of CT of soldiers including cadets. AA seems to be highly important in achieving high shooting results because a shooter is forced to switch his attention between individual elements important in effective SA (stance, aiming, alignment of aiming devices (bow and pin barrel), simultaneous focus on the target and aiming devices, breathing, pulling the trigger and firing the shot). The results of our study indicate that there is a relationship between the level of attention and SA. Thus, AA may be regarded as a strong determinant of cadets’ SA.

However, it seems that other processes related to attention and executive functions, i.e., intentional search of material, sustained attention, sequential processing of information, and self-monitoring, may also be important for high SA. The ability to deliberately and actively select specific data from the environment in shooting comes down to focusing attention only on selected important information (shooting actions, weather conditions, target, weapon) while ignoring other distracting stimuli from the environment (e.g., other shooters and sounds of shots, other external sounds, environment, time) [38].

Maintaining attention in shooting is mainly for focusing on the target for as long as possible. It requires full commitment and concentration on the target. In practice, in case of fatigue or reduced attention, a technique is used in which one stops aiming for a few seconds, directing the gaze to an object of green color, and then returns to aiming with full focus and concentration [39].

Sequential processing of information in shooting is based on the analysis of all the elements in turn, capturing the interrelationships between them, ultimately combined as a set of actions performed to make an accurate shot, e.g., combining the appropriate rhythm of breathing with the simultaneous pulling of the trigger [40].

Monitoring one’s own shooting behavior is primarily about controlling emotions. While shooting, one should remain fully focused and calm. Anxiety or stress translates into increased heart rate, increased respiratory rhythm or even muscle tension, which significantly hinders small motor control in shooting activities [41]. Among other things, research confirms that mental fatigue can impair small motor control and affect how shooters respond to targets [42] and the results they achieve [43]. Furthermore, other studies have shown that subtle disruptions in the attention process can lead to dramatic changes in performance [44,45].

Despite the lack of similar studies on the relationship between AA and level of CT, mainly cadet SA, our results correspond with other studies in archery [46] and sport shooting athletes [16]. The results of these studies provided preliminary evidence for the importance of interoceptive attention in shooting sports and archery [46], and significant narrowing of attention and higher levels of alertness were observed in experienced pistol shooters [16]. However, comparisons with self-reported studies should be made with caution because there are important differences in shooting conditions. Both in archery and in sport shooting there is a different type of weapon and, moreover, shooting is performed in sporting attire, without additional loads such as vest, tactical equipment or helmet.

It seems that the results of our study add to the knowledge concerning the role of attention and concentration in achieving high results in shooting. In fact, earlier studies focused on a group of professional soldiers: marksmen [15] and expert and novice infantry soldiers [41]. It turns out that regardless of the degree of shooting proficiency and the level of CT, attention span and concentration are equally important in a group of cadets (own research), preparing for professional service.

Taking into account the above literature reports, the conditions of the research, its conclusions and the results of our own research it may be concluded that in static shooting in standing and lying postures AA is of particular importance for SA. Therefore, it is necessary to continue the research on the influence of attention-related processes on SA in order to precisely determine which parts of attention have the most significant influence on shooting results in different shooting stances and tasks.

## 6. Limitations, Strengths and Future Research

The study was conducted among a highly selected group of individuals in terms of PF and academic achievement. Therefore, the results of the study should not be generalized to all civilian and military students. It should also be noted that the measure of cadets’ CT in the present study was SA and academic achievement in specific PMS, and did not take into account other components of this training such as medical training, communications, leadership, experience, among others. It is also difficult to directly examine the level of one component of attention because existing neuropsychological tests require the subject to integrate cognitive functions with executive functions (switching, inhibition, working memory) [47].

Since there is a lack of research in the literature on the relationship between AA and PF and cadet CT, this opens up useful new areas of research, especially for researchers in the military community. Other factors (e.g., soldiers’ level of motivation or physical activity) potentially important to CT levels would be worth considering in future similar studies. In addition, a lot of valuable information may be provided by the results of future studies on the effectiveness of dynamic shooting under fatigue conditions, as they will more closely match the conditions of combat operations.

## 7. Conclusions

The level of CT of the soldier (and especially the commander–leader) is of great importance to the armed forces in national defense and should be a key aspiration of every military unit. The readiness of each soldier for warfare is inextricably linked to the level of physical and cognitive fitness. The results of conducted research suggest the necessity of placing great emphasis on the physical preparation of modern soldier, especially focused on the development of strength and endurance capabilities, which have the greatest impact on the level of CT of the soldier. These abilities to the greatest extent reflect the requirements of combat tasks on the battlefield.

Due to the role of effective shooting, the authors also indicate the relevance of introducing cognitive stimulation exercises such as active-response-inhibition training [48] or mindfulness-based attention training [49] to shooting training. This knowledge should be of interest to researchers in the uniformed services community and those professional groups in which weapons are the primary tool of work.

## Figures and Tables

**Table 1:** Descriptive statistics of the study variables.

Variable	*n*	$\bar{x}$	Reliance −95.0%	Reliance 95.0%	sd	v
Grip strength	137	131.50	128.32	134.67	18.80	14.30
Shuttle run 10 × 5 m		17.98	17.78	18.18	1.20	6.66
Distance run 1000 m		211.42	207.49	215.34	23.23	10.99
Color Trails Test (CTT-2)		59.84	57.72	61.96	12.54	20.95
Academic achievements in PMS		4.17	4.11	4.24	0.38	9.19
Shooting accuracy		35.58	34.60	36.56	5.81	16.34

$\bar{x}$ —mean, sd—standard deviation, v—coefficient of variation.

**Table 2:** Pearson correlations between the analyzed variables.

Variable	Shooting Accuracy	Academic Achievements in PMS
CTT-2	**−0.57**	−0.07
Grip strength	0.06	**0.18**
Shuttle run 10 × 5 m	−0.05	0.06
Distance run 1000 m	0.15	**−0.24**

Correlation coefficients highlighted in bold are significant with *p* < 0.05.

**Table 3:** Results of regression analysis (progressive stepwise method) of cadets’ shooting accuracy (SA) as a function of their level of alternating attention (AA) and physical fitness (PF).

Test for Full Model						Standardized Coefficients β for Selected Variables
Variable	F	*p*	Adjusted R^2^	SEE	B_0_	CTT-2
Shooting accuracy	**63.49**	**0.0000**	**0.315**	**0.071**	**51.27**	**−0.566**

Adjusted R^2^—coefficient of determination, SEE—standard error of estimation, B_0_—value of the constant, β significant at the *p* < 0.05 level—is indicated in bold.

## Data Availability

The datasets used and/or analyzed during this study are available from the corresponding author on reasonable request.
